# Sudden Death in a Child Revealing a Multifocal Cardiac Fibroma: A Hidden Lethal Threat

**DOI:** 10.7759/cureus.98126

**Published:** 2025-11-30

**Authors:** Achref Miry, Mohammed Tbouda, Kenza Oqbani, Soulaymane Dachi, Sanae Abbaoui

**Affiliations:** 1 Pathology and Laboratory Medicine, Faculty of Medicine and Pharmacy of Agadir, Agadir, MAR; 2 Pathology, Souss Massa University Hospital, Agadir, MAR; 3 Pathology, Oued Eddahab Military Hospital, Agadir, MAR; 4 Pathology, Medical and Pharmacological Faculty, Ibn Zohr University, Agadir, MAR; 5 Pathology, Faculty of Medicine and Pharmacy of Agadir, Agadir, MAR

**Keywords:** cardiac fibroma, fibrous neoplasm, multifocal cardiac lesion, pediatric cardiac tumor, sudden death

## Abstract

Despite their benign histology, cardiac fibromas can cause life-threatening complications, including arrhythmias and sudden death. We report the case of a previously healthy four-year-old boy who collapsed suddenly after a minor fall. Despite immediate resuscitation efforts, he was declared dead. Autopsy revealed an enlarged heart with a large, firm, white intramyocardial mass measuring 5.5 × 4.5 × 3.0 cm in the lateral wall of the left ventricle, compressing the ventricular cavity. Three additional epicardial nodules were also identified. Histologic examination showed spindle cell proliferation with dense collagenous stroma, low mitotic activity, and no atypia or necrosis, consistent with multifocal cardiac fibroma. This case illustrates the silent yet potentially lethal nature of cardiac fibromas in children. Multifocal presentation is extremely rare and can remain undiagnosed until sudden death occurs. Awareness of such tumors and early cardiac imaging may help prevent fatal outcomes in pediatric populations.

## Introduction

Primary cardiac tumors in children are exceptionally rare, accounting for less than 0.3% of all cardiac tumors [1]. Among them, cardiac fibroma is the second most common benign entity after rhabdomyoma, typically arising in the left ventricular free wall or the interventricular septum [2]. Although histologically benign, fibromas can lead to severe complications such as ventricular arrhythmias, obstruction of cardiac chambers, or sudden death [1,3]. Despite advances in noninvasive cardiac imaging, especially echocardiography and cardiac magnetic resonance imaging, many cases remain asymptomatic and undetected until a fatal outcome occurs [3,4]. We report the case of a four-year-old boy who died suddenly following a syncopal episode, with postmortem findings revealing a large intramyocardial fibroma of the left ventricle, illustrating the lethal potential of these tumors even in previously healthy children.

## Case presentation

A four-year-old previously healthy boy suddenly lost consciousness following a low-energy fall from standing height. The episode was immediately followed by cardiorespiratory arrest. Despite the arrival of emergency medical services, resuscitation was unsuccessful, and the child was declared dead. There was no reported history of prior cardiac symptoms such as palpitations, syncope, chest pain, or reduced exercise tolerance.

**Figure 1:**
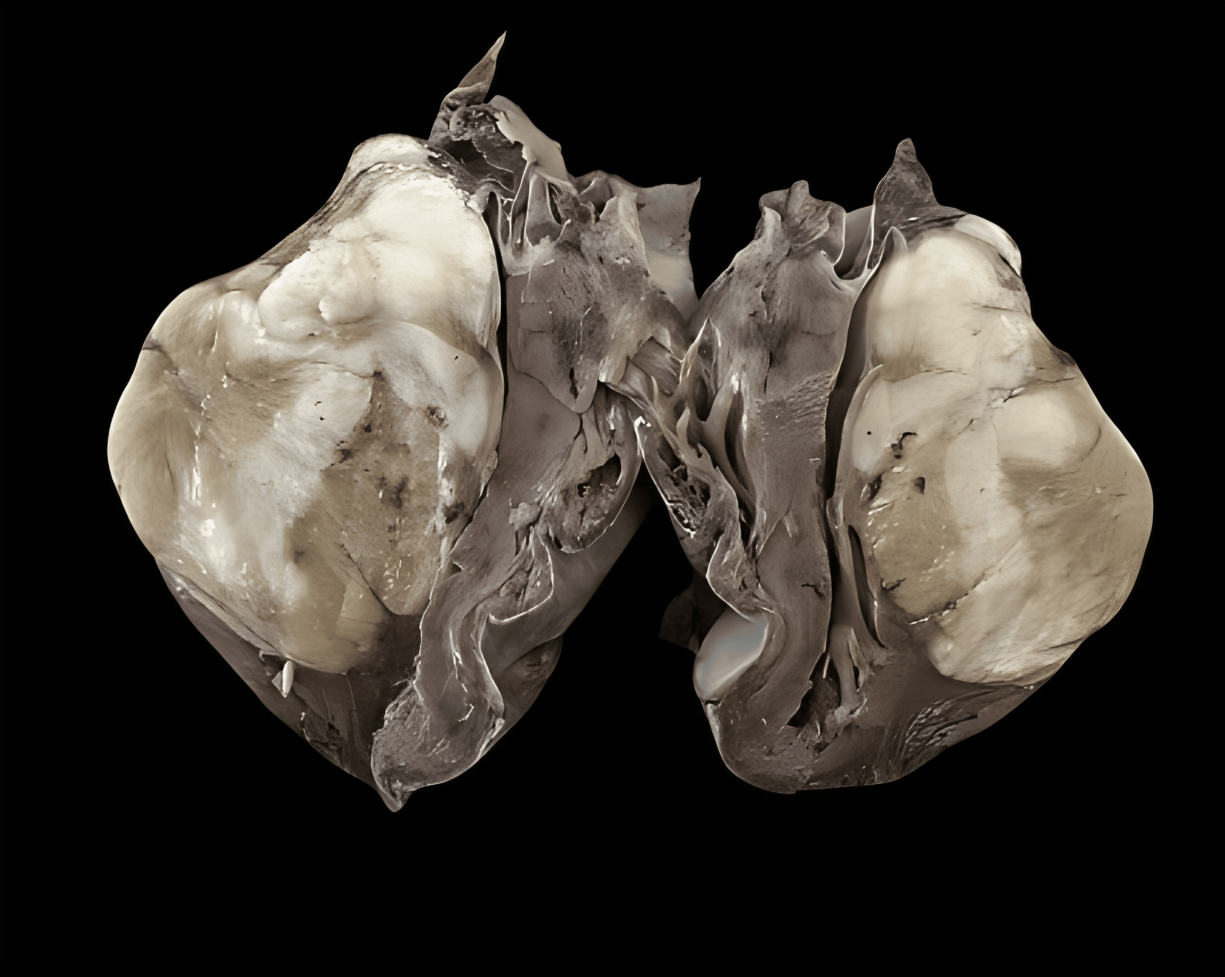
Cut a section through the mass showing a firm, homogeneous, white surface without necrosis or hemorrhage.

Autopsy revealed a markedly enlarged heart, weighing 213 grams and measuring 9 × 6 × 6 cm (normal average for age: approximately 85-100 g). The pericardial sac was intact and free of effusion. A well-demarcated intramyocardial mass was discovered within the lateral wall of the left ventricle, protruding into the ventricular cavity. The lesion measured 5.5 × 4.5 × 3.0 cm and was firm, white, and homogeneous on the cut section, without macroscopic hemorrhage or necrosis. The mass extended from the mid-lateral ventricular wall to the atrioventricular junction. No evidence of valvular involvement or thrombi was observed. The left ventricular cavity appeared collapsed and compressed by the tumor mass (Figure 1).

**Figure 2:**
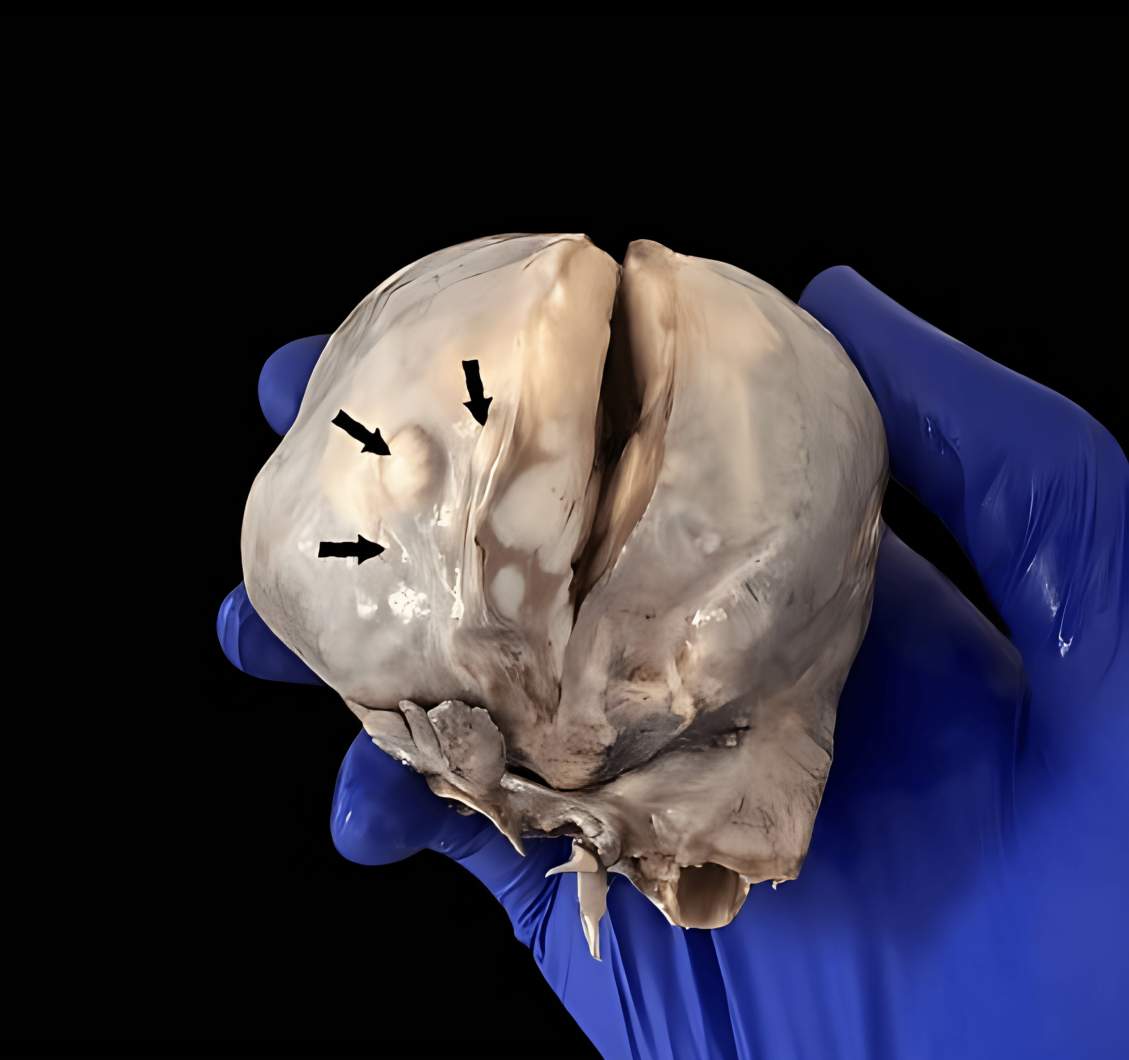
Photograph showing three small satellite lesions with the same macroscopic appearance as the main lesion (arrows).

In addition, three discrete nodules were observed on the epicardial surface: two subcentimetric nodules measuring 2 mm each, and one measuring 9 mm. These nodules shared the same gross features as the primary tumor (Figure 2).

**Figure 3:**
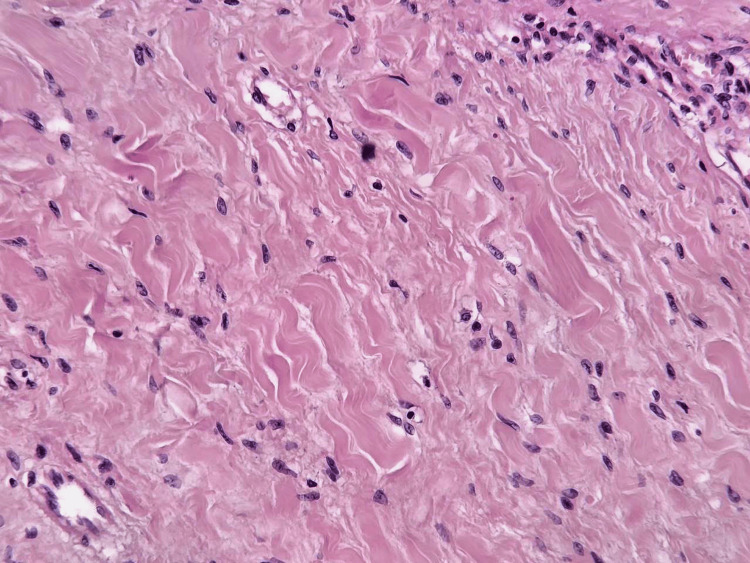
Spindle cells arranged in fascicles within dense collagenous stroma (H&E, ×100).

**Figure 4:**
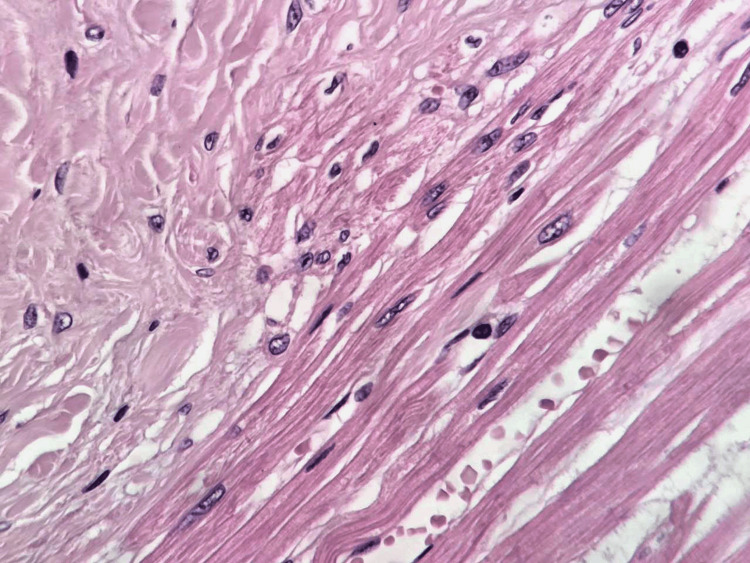
High-power view showing bland nuclei, rare mitoses, and absence of atypia (on the left); normal cardiac myocytes with cross striations are observed on the right (H&E, ×400).

Microscopic analysis of the ventricular mass demonstrated a proliferation of bland spindle-shaped cells with elongated, uniform nuclei and scant eosinophilic cytoplasm, arranged in long intersecting fascicles. The stroma was dense and fibrocollagenous, with thick collagen bundles and no myxoid or hemorrhagic areas (Figure 3). Tumor cellularity was moderate to low, with occasional hypocellular zones. Mitoses were rare (<1/10 high-power fields), and no atypical mitotic figures were seen (Figure 4). 

**Figure 5:**
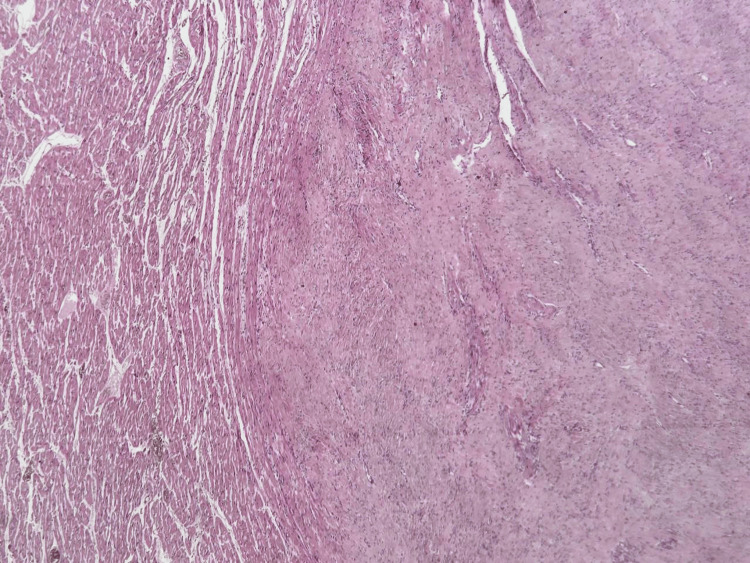
Low-power view showing a well-demarcated intramyocardial tumor with entrapped myocardial fibers (H&E, ×40).

**Figure 6:**
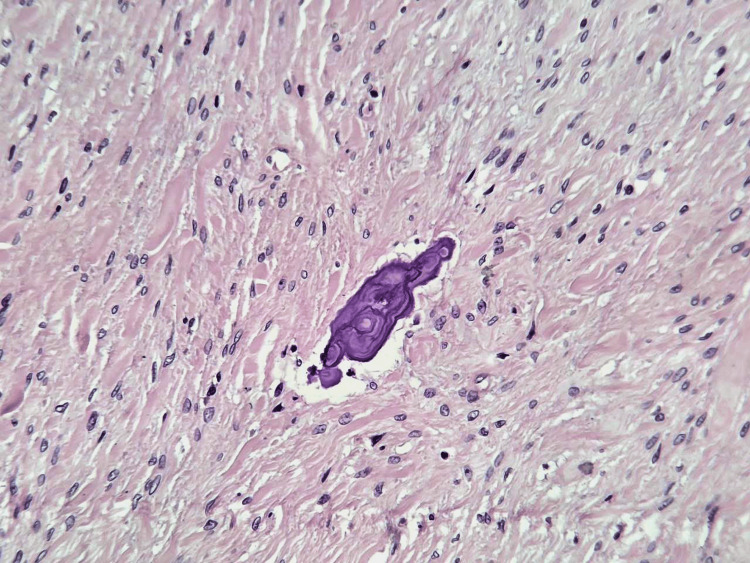
Dystrophic calcifications within fibrous stroma (H&E, ×200).

The tumor was non-encapsulated but clearly demarcated from the adjacent myocardium (Figure 5), which was sometimes entrapped within the lesion. No evidence of perivascular invasion or myocardial destruction was observed. Importantly, focal dystrophic calcifications were noted within the collagen matrix (Figure 6). There was no associated inflammatory infiltrate, necrosis, or areas of cystic degeneration.

Histologic sections from the epicardial nodules revealed the same spindle cell proliferation, confirming their nature as satellite lesions of the main fibroma.

The overall histomorphologic features were consistent with multifocal cardiac fibroma.

## Discussion

Cardiac fibroma is a rare but significant primary tumor of the pediatric heart. Although histologically benign, its clinical behavior may be unpredictable and potentially life-threatening, depending on size, location, and interaction with vital cardiac structures. In this case, the tumor remained clinically silent until a fatal syncopal episode, emphasizing the importance of awareness and early detection in pediatric populations.

Epidemiology and pathogenesis

Cardiac tumors in children are rare, with an estimated incidence ranging from 0.0017% to 0.28% in autopsy and imaging studies [1]. Among benign tumors, rhabdomyoma is the most common, particularly in infants, followed by fibroma, which accounts for 6-25% of primary cardiac tumors in children [1,2,4]. Cardiac fibromas arise from fibroblasts of the myocardial connective tissue, and unlike rhabdomyomas, they do not regress spontaneously and tend to grow progressively [4,5].

Fibromas typically occur as solitary intramural lesions, most often in the left ventricular free wall or the interventricular septum [2,4]. Their pathogenesis remains incompletely understood, though associations with genetic syndromes such as Gorlin syndrome (nevoid basal cell carcinoma syndrome) have been described in rare cases [4,5].

Clinical presentation

The clinical manifestations of cardiac fibroma depend primarily on the tumor’s size and anatomic location. While some tumors remain asymptomatic, others may cause a wide spectrum of symptoms, including murmur, dyspnea, chest pain, palpitations, arrhythmias, syncope, or even sudden cardiac death [2,3,4,6].

In a study of 166 pediatric cardiac tumors, ventricular arrhythmias were reported in up to 64% of fibroma cases, especially when the tumor involved the conduction system [3]. The risk of sudden death increases in cases of large fibromas compressing the ventricular cavity, obstructing outflow tracts, or infiltrating critical conduction pathways [2,4,6]. In our case, the child had no prior symptoms, and the tumor remained undiagnosed until the fatal event, a clinical pattern described in several postmortem studies of pediatric sudden death [1,2,4,6,7].

Imaging and diagnosis

Modern cardiac imaging techniques play a central role in the detection and characterization of cardiac tumors. Transthoracic echocardiography remains the first-line investigation due to its accessibility and high sensitivity [4,6]. However, cardiac magnetic resonance imaging (CMR) provides superior tissue characterization, allowing assessment of tumor margins, tissue composition, enhancement patterns, and relation to coronary arteries or conduction tissue [3,4,8].

In the case reported by Panaioli et al., CMR revealed a well-defined left ventricular fibroma with avid contrast enhancement, while CT confirmed the intratumoral course of the left anterior descending artery [3]. Such imaging findings are crucial for surgical planning and risk stratification. Unfortunately, in our case, no imaging was performed ante-mortem, highlighting the diagnostic challenge in asymptomatic children.

Histopathology

Cardiac fibromas are characterized by bland spindle-shaped fibroblasts arranged in fascicles within dense collagenous stroma. They are typically non-encapsulated but well delineated and may exhibit foci of calcification or cystic degeneration in large lesions [4-6]. Mitoses are rare, and nuclear atypia is absent. These features help distinguish fibromas from malignant soft tissue tumors, such as fibrosarcomas or undifferentiated sarcomas.

Our case displayed classic histopathological findings consistent with fibroma: a non-encapsulated spindle cell proliferation with moderate to low cellularity, absence of atypia, and focal dystrophic calcifications. The presence of epicardial satellite nodules of similar morphology is rare and may reflect multifocal growth or local extension.

Prognosis and management

Despite their benign nature, cardiac fibromas may carry a poor prognosis if untreated, particularly when associated with intractable arrhythmias or hemodynamic compromise. Surgical resection remains the treatment of choice in symptomatic patients and may lead to full recovery of cardiac function [3,4,6]. However, in cases where resection is not feasible due to involvement of vital structures (e.g., coronary arteries), cardiac transplantation may be considered [4,5].

Asymptomatic patients with small fibromas may be followed conservatively with serial imaging. However, the threshold for surgical intervention remains low, especially in cases with arrhythmias, syncope, or signs of compression [4,6].

Sudden death in children

Sudden death due to cardiac tumors is a rare but documented phenomenon in pediatric forensic pathology. Several autopsy series have revealed undiagnosed cardiac fibromas as the cause of sudden unexplained death in previously healthy children [2,4,6,7]. Mechanisms include arrhythmic death due to involvement of the conduction system, mechanical obstruction of blood flow, or myocardial ischemia secondary to coronary compression.

In the present case, the child’s sudden death following minimal trauma and the presence of a large left ventricular mass compressing the cavity strongly suggest a fatal arrhythmic or obstructive mechanism.

Multifocal cardiac fibromas

Cardiac fibromas are typically solitary lesions; however, multifocal presentations, as observed in our case with associated epicardial nodules, are exceedingly rare. Multifocal fibromas may result from satellite spread along the epicardial surface or represent multicentric origin from embryonic mesenchyme. Only isolated reports in the literature describe such presentations, and their clinical significance remains uncertain [9]. Some authors hypothesize that the presence of multiple nodules may reflect local myocardial dissemination or developmental anomalies during cardiac morphogenesis. Importantly, multifocality has not been associated with increased malignancy risk but may complicate complete surgical excision and increase the risk of arrhythmic complications [9,10].

Genetic predisposition and syndromic associations

Although most cardiac fibromas are sporadic, a minority may occur in association with genetic syndromes. The most well-known is Gorlin syndrome, also known as nevoid basal cell carcinoma syndrome, an autosomal dominant disorder caused by pathogenic variants in the PTCH1 gene on chromosome 9q22.3. This syndrome is characterized by multiple basal cell carcinomas, odontogenic keratocysts, and skeletal abnormalities, but cardiac fibromas have also been reported in approximately 3-5% of cases [4,11]. Recognition of this association is critical for patient and family counseling, as well as long-term surveillance. Other syndromes, such as Tuberous Sclerosis Complex (TSC), are more commonly linked to rhabdomyomas, but isolated reports of fibroma-like lesions have occasionally been described in TSC patients, suggesting possible phenotypic overlap [12].

In all cases of pediatric cardiac tumors, especially when multifocality or extracardiac anomalies are present, a thorough clinical evaluation for syndromic features and referral for genetic counseling should be considered.

## Conclusions

Cardiac fibromas in children, while histologically benign, may cause fatal outcomes due to arrhythmia or mechanical obstruction. The multifocal presentation observed in this case is exceptionally rare and emphasizes the importance of considering cardiac tumors in unexplained pediatric sudden death. Early recognition through imaging and awareness of such entities is crucial to improving prevention and management.
